# Increased serum levels of lipogenic enzymes in patients with severe liver steatosis

**DOI:** 10.1186/1476-511X-11-145

**Published:** 2012-10-30

**Authors:** Maria Notarnicola, Giovanni Misciagna, Valeria Tutino, Marisa Chiloiro, Alberto Ruben Osella, Vito Guerra, Caterina Bonfiglio, Maria Gabriella Caruso

**Affiliations:** 1 Laboratory of Biochemistry, National Institute for Digestive Diseases, Castellana Grotte, 70013, Bari, Italy; 2 Laboratory of Epidemiology and Biostatistics, National Institute for Digestive Diseases, Castellana Grotte, Bari, Italy; 3 Division of Radiology, National Institute for Digestive Diseases, Castellana Grotte, Bari, Italy

**Keywords:** Fatty acid synthase, Lipoprotein lipase, Liver steatosis, Enzyme activity

## Abstract

**Background:**

Lipid metabolism is altered in subjects with liver steatosis. FAS is a key enzyme in *de novo* lipogenesis and both FAS gene expression and enzymatic activity are primarily regulated by metabolic signals in the liver. Lipoprotein lipase (LPL), the rate-limiting enzyme for the hydrolysis of core triglycerides, plays a pivotal role in lipid metabolism. This study aims to investigate if circulating levels of FAS and LPL could be clinically associated with liver steatosis.

**Methods:**

In this work, we present data obtained from a subsample of 94 subjects with liver steatosis enrolled by NUTRIEPA study, a nutritional trial in subjects with liver steatosis. Serum levels of FAS protein and LPL activity were evaluated by ELISA test and by a fluorescent method, respectively. The diagnosis and the degree of liver steatosis were based on laboratory and ecographic measurements. Statistical methods included Kruskal-Wallis analysis of variance and Wilcoxon signed-rank test, where appropriate. The *χ*^2^ test has been performed to analyse categorical variables.

**Results:**

The subjects with severe steatosis had significantly higher serum levels of FAS protein and LPL activity compared to subjects with mild and moderate liver steatosis. Moreover, a positive trend in serum levels of FAS expression from lower to higher degree of steatosis was also detected.

**Conclusions:**

We describe a relationship between human liver steatosis and elevated levels of circulating lipogenic enzymes. Increased serum levels of FAS expression and LPL activity could be considered a marker of severe liver steatosis.

## Introduction

Liver steatosis may be considered the consequence of an increased influx of free fatty acids or a decreased export of lipids through very low density lipoprotein (VLDL), less fatty acid oxidation or increased de novo lipogenesis in the liver [1]. Liver steatosis is frequent in patients with metabolic syndrome and accumulating evidences suggest that lipid metabolism is as important to diabetes as carbohydrate metabolism [2].

Insulin is the most lipogenic hormone [2,3] and it is an important regulator of fatty acid synthase (FAS). FAS is a cytosolic multi-enzyme that functions normally in the liver and is minimally expressed in other tissues [4]. FAS is a key enzyme in *de novo* lipogenesis [4,5] and both FAS gene expression and enzymatic activity are primarily regulated by metabolic signals in the liver [3]. Despite being an intracellular protein, it may be released into the extracellular space and may be a biomarker of metabolically demanding human diseases [6]. Increased FAS levels have been detected in serum of patients with different clinical stages of breast cancer [7]. Recently, a significant association between circulating levels of FAS and HER2-overexpressing metastatic breast cancer patients has been described [8]. Moreover, our previous study showed that serum levels of FAS in colorectal cancer patients were associated with tumor stage, suggesting that serum FAS detection can be used for distinguishing the patients with metastatic colorectal cancer [9]. Recently, high serum levels of FAS have been detected in patients with chronic hepatitis viral infections and circulating FAS concentration correlated with the degree of liver steatosis [10].

Enhanced FAS expression is a poor prognostic factor in patients with breast, prostate, and ovarian cancer, generating an intriguing paradigm that cancers with high expression of FAS have a growth and/or survival advantage [11,12]. FAS expression appears also to play an important role in the growth and neoplastic transformation of colonic mucosa [12,13]. Our previous study demonstrated that FAS activity levels, as well as the expression of its mRNA are up-regulated in colorectal cancer tissues compared with “normal” surrounding mucosa [13].

Lipoprotein lipase is the rate-limiting enzyme for the hydrolysis of core triglycerides in chylomicrons and VLDL [14]. LPL plays a pivotal role in lipid metabolism and changes in LPL expression could affect both the rate of fat accumulation and the metabolism of triglyceride-rich lipoproteins [15]. In patients with resectable non-small cell lung cancer, high levels of LPL activity have been detected in cancer tissue [16]. Moreover, increased LPL activity in non-small cell lung cancer tissue predicts shorter patient survival, independent of standard prognostic factors. Recently, we have demonstrated a significant reduction in both LPL and FAS gene expression and activity levels in peritumoral adipose tissue of colorectal cancer patients [17], demonstrating that a tumor-induced impairment in the formation and lipid storing capacity of adipose tissue occurs in patients with colorectal cancer.

A recent study shows that an up-regulation of LPL evoked an insulin resistant state in hepatocytes in mice and finally developed into hepatic steatosis [18].

On the basis of these evidences, the measurement of circulating FAS and LPL could be useful in the management of metabolic diseases, as steatosis. This study aims to explore serum levels of FAS protein and LPL activity in patients with steatosis in order to assess a possible clinical association between levels of enzyme expression and degree of liver steatosis.

## Methods

### Patients

This work is part of NUTRIEPA study, a nutritional trial enrolling consecutive subjects with liver steatosis, diagnosed by abdominal ultrasound.

In this report, we present data obtained from a subsample of 94 subjects. All subjects underwent a complete medical examination including weight and height measurements. Body mass index (BMI) was calculated as weight in kilograms divided by the square of the height in meters (kg/m^2^). Participants were fasted for 12 h prior to examination. Blood samples taken from the subjects by venous puncture were collected in tubes containing a serum separator gel. Aliquotes of blood serum were stored at −80°C until assayed or shipped to the central laboratory for routine analyses. All the analyses were performed within 6 months.

In order to obtain a semiquantitative evaluation of fat in the liver, we used a system scoring [19,20]. Degree of fatty infiltration of the liver was graded according to ultrasonographic appearance of liver echotexture, hepatic echo penetration and clarity of hepatic blood vessels, liver diaphragm differentiation in echo amplitude. Each criterion was assigned a score indicating the level of fatty liver infiltration. A score of 2 indicated a definite positive (++) fatty liver infiltration for that criterion. A score of 1 was assigned for a probable positive (+) finding based on the criterion. When a negative (−) evaluation for fatty liver was made from a criterion, a score of 0 was assigned to the criterion. The sum of the scores for the three criteria was considered to be an indicator of severity of fatty infiltration. Thus, the fatty liver indicator ranged from 0 to 6, with mild fat infiltration for a total score 1–2; moderate for a total score of 2–4, severe indicated by a total score ≥ 5.

Moreover, a control group of subjects without liver steatosis was included. In this group, the liver steatosis was excluded by ultrasonographic and laboratory evaluation.

The study had institutional approval and all patients gave informed consent to participate in the study.

### FAS analysis

FAS analysis was performed by a commercially available ELISA kit, FAS-detect ELISA (FASgen, IMMTECH, Baltimore, MA, USA) according to the manufacturer's recommendations.

Briefly, sera were incubated in a 96-well capture plate on a plate shaker for 90 min at room temperature. The plate was then washed five times with wash buffer. FAS enzyme conjugate was added and the plate was incubated for 60 min, and the wash was repeated. Serum FAS levels were visualized by color change upon addition of tetramethyl-benzidine substrate followed by addition of substrate stop solution. Absorbance values were read at 450 nm using a spectrophotometer. FAS concentrations were determined by interpolation from the standard curve. Intra-and inter-assays coefficients of variation (CV) were less than 8%, and the detection limit was 2.0 ng/ml.

### LPL analysis

LPL enzymatic activity was evaluated on serum samples by a fluorescent method (LPL activity Kit, Roar Biomedical, New York, NY). Aliquotes of 100 μl of serum were incubated with 100 μl of pre-diluited substrate emulsion at 37°C for 1 h, according to the manufacturer's recommendations. The hydrolyzed triglycerides formed were measured at 370 nm excitation and 450 nm emission. The fluorescence intensity values of samples were compared with the fluorescence intensity values of standard curve applied on the same plate together with samples in each run. LPL activity was expressed as picomoles of hydrolyzed substrate per minute per ml of serum (pmol/min/ml). All measurements were made in duplicate and intra-and inter-assays coefficients of variation (CV) were less than 5%.

### Laboratory measurements

In order to estimate the HOMA index, serum levels of glucose and insulin were measured by an automated system (Uni Cel Integrated Workstations DxC 660i, Beckman Coulter, Fullerton, California, USA) using chemioluminescent immunometric methods. Samples over calibration range were automatically reanalyzed according to constructor's instructions.

### Statistical analysis

The significance of the differences among the groups was evaluated by Kruskal-Wallis analysis of variance, Wilcoxon signed-rank or Mann–Whitney test, where appropriate, and *χ*^2^ test for trend. All data were expressed as mean ± SD. Differences were considered significant at *P* < 0.05. The *χ*^2^ test has been performed to analyse categorical variables.

## Results

Liver steatosis was diagnosed and its grade was assessed with the findings of liver ultrasound scan performed by the same radiologist. We found 33 subjects with mild liver steatosis, 38 with moderate and 23 with severe steatosis.

Table 1 shows descriptive analysis of studied variables in controls and subjects stratified for degree of liver steatosis.

**Table 1:** Descriptive analysis of the studied variables in controls and in subjects with liver steatosis

	**Controls (n=21)**	**Mild Steatosis (n=33)**	**Moderate Steatosis (n=38)**	**Severe Steatosis (n=23)**	***P*value⁁°**	**Contrasts*P*value**^**§**^
Sex					0.36	
*male (%)*	12 (57.2%)	6 (18.2)	9 (23.7)	8 (34.8)		
*female (%)*	9 (42.8%)	27 (81.8)	29 (76.3)	15 (65.2)		
Age (years)	44.2±10.5	54.9±10.7	53.1±12.3	49.8±11.5	0.37	
BMI (kg/m^2^)	25.2±4.1	32.1±3.3	31.6±4.8	34.7±5.4	0.02	Mild *vs* Severe
					0.001*	*P*= 0.03
						Moderate *vs* Severe *P*= 0.01
HOMA index	1.3±0.9	3.2±1.5	3.5±1.9	7.03±14.0	0.04	Mild *vs* Severe
					0.001*	*P*= 0.018
						Moderate *vs* Severe *P*= 0.07
FAS (ng/ml)	10.6±7.6	12.4±17.2	19.3±15.3	22.1±20.3	0.005	Mild *vs* Moderate
					0.02*	*P*= 0.007
						Mild *vs* Severe
						*P*= 0.004
						Severe *vs* Controls
						*P*= 0.005
LPL activity (pmol/min/ml)	29.4±2.6	34.1±4.8	36.6±3.0	65.1±8.3	0.001	Controls *vs* Severe
					0.07*	*P*= 0.003
						Mild *vs* Severe
						*P*= 0.002
						Moderate *vs* Severe
						*P*= 0.003

All data expressed as percentage or M±SD; ⁁*χ*^2^-square test for categorical variable; °Kruskal-Wallis test for continuous variable; ^§^Wilcoxon rank-sum (or Mann–Whitney) test; *Test for trend.

Serum levels of FAS protein and LPL activity among the groups were significantly different. The subjects with steatosis showed circulating levels of FAS and LPL significantly higher than controls. Moreover, stratifying for score of steatosis, the subjects with severe steatosis had significantly higher serum levels of FAS protein and LPL activity compared to subjects with mild and moderate liver steatosis.

In addition, a positive and statistically significant trend in serum FAS concentration from lower to higher degree of steatosis was also detected (Table 1).

The values of BMI and HOMA index were statistically different among the groups of subjects and the test for trend was also significant (Table 1).

No difference among the groups was detected in relation to sex and age (Table 1).

## Discussion

In this study we describe a relationship between human liver steatosis and elevated levels of circulating lipogenic enzymes expression, such as FAS and LPL. To our knowledge, we show for the first time, elevated levels of LPL activity in serum from patients with severe steatosis, suggesting that serum LPL activity measurement may add substantial information for the evaluation of liver impairment.

The over-expression of FAS is associated with the degree of liver steatosis because of a positive trend was detected in serum levels of FAS from lower to higher degree of steatosis.

Although liver biopsies and nuclear magnetic resonance imaging are the methods of choice for diagnosis of liver steatosis, ultrasonography is currently the preferred method for screening asymptomatic patients with suspected liver injury [21]. Several studies have demonstrated that the sensitivity, specificity and positive predictive value of this technique to detect steatosis as high as 80-100% [22-24]. Taking into account for the ethical concerns, we could not perform liver biopsy of our patients. However, in the light of clinical and laboratory parameters, the diagnosis of steatosis was clearly demonstrated.

FAS and LPL are central enzymes of lipid metabolism. FAS is believed to be a major determinant of the maximal hepatic capacity to generate fatty acids by *de novo* lipogenesis [5]. Over-expression of FAS has been detected in non-alcoholic fatty liver disease (NAFLD) where increased mitochondrial oxidation of fatty acids takes place [4]. FAS expression in steatosic hepatocytes may be considered as a compensatory adaptation at early stages of NAFLD.

The state of over-nutrition-induced insulin resistance and decreased *de novo* lipogenesis have been demonstrated to be associated with increased FAS in serum [2,25]. Circulating FAS levels of obese individuals could be considered a marker of metabolic failure in the contex of over-nutrition-induced insulin resistance and metabolic stress [25]. A recent study demonstrates enhanced serum levels of FAS in patients with chronic hepatitis viral infections and they correlated with the degree of liver steatosis [10].

Dysfunction of LPL has been indicated in several disorders including dyslipidemia and atherosclerosis [26]. Sequenzial changes in the activities of LPL and lipogenic enzymes have been demonstrated in patients with tumor, as well as in animal model of cancer [16,17,27,28]. Moreover, hepatic LPL deficiency seems to protect against diet-induced obesity and hepatic steatosis in mice [18].

LPL generates free fatty acids that induce insulin resistance in muscle and liver [29]. It has been suggested that insulin resistance is an essential requirement for the accumulation of hepatocellular fat [30].

In this study, high levels of FAS and LPL activity detected in subjects with steatosis are in agreement with the presence of insulin resistance evaluated in same subjects. Moreover, the patients with severe liver steatosis showed a value of BMI significantly higher, demonstrating that some individuals develop metabolic complications in relation to their degree of obesity.

## Conclusions

FAS and LPL, intracellular proteins, appear to exit cells during liver impairment and their presence in serum could be considered a marker of liver steatosis.

Therefore, it is clinically important that specific biomarkers of metabolic changes are identified in order to assess the efficacy of diagnosis and therapeutic treatment.

## Competing interests

The authors declare that they have no competing interests.

## Author’s contributions

MN conceived the study, interpreted the data and wrote the manuscript; GM participated in critical revision of study; VT performed experiments; MC performed ecographic measurements; ARO and CB participated in patient enrolement; VG performed statistical analysis; MGC conceived the study, participated in its design. All authors read and approved the final manuscript.
